# Screening High-Risk Groups and the General Population for SARS-CoV-2 Nucleic Acids in a Mobile Biosafety Laboratory

**DOI:** 10.3389/fpubh.2021.708476

**Published:** 2021-08-13

**Authors:** Zhimin Guo, Lin Li, Yuanyuan Song, Jiancheng Xu, Jing Huang

**Affiliations:** Department of Clinical Laboratory, The First Hospital of Jilin University, Changchun, China

**Keywords:** SARS-CoV-2, nucleic acid, real-time PCR, bio-protection, mobile biosafety laboratory

## Abstract

The Severe Acute Respiratory Syndrome coronavirus 2 (SARS-CoV-2) pandemic has challenged public health systems worldwide. Therefore, large-scale testing capacity is extremely important diagnosis and exclusion diagnosis. However, fixed laboratories are limited or far away from remote areas. Fortunately, MBS-Lab is characterized by high mobility and rapid on-site detection of SARS-CoV-2 nucleic acid. MBS-Lab was first used in northern Australia during a melioidosis outbreak in 1997. The MBS-Lab and a well-trained diagnostic team were dispatched to Dongchang District, Tonghua City, Jilin Province, China to assist the SARS-CoV-2 virus screening and diagnosis on January 17, 2021. Altogether, 93,952 oropharyngeal swabs samples were collected and tested among the high-risk groups and the general population in Dongchang District. Two single samples were identified as positive in the second turn screening. In the second turn screening, 3 mixed samples (10 in 1) were identified as positive; 10 mixed samples were identified as positive in the third turn screening. By resampling again, one and four cases were identified as positive, respectively. The positive cases were properly isolated and treated in hospital and avoided to visit family members, friends, colleagues and any other persons. Through this way of large-scale screening, human-human spread of SARS-CoV-2 can be effectively avoided. In addition, all staff members strictly executed multiple safety precautions and reduce exposure risks. In the end, none of the staffs was infected with SARS-CoV-2 virus or other pathogens. As an emergency facility for infectious disease control, the MBS-Lab satisfies the requirements of ports and other remote areas far from fixed laboratories and supplements the capabilities of fixed laboratories.

## Introduction

The severe acute respiratory syndrome coronavirus 2 (SARS-CoV-2) has challenged public health systems worldwide. As of 21 June 2021, the total number of cases worldwide is over 178 million and the total number of deaths is 3.8 million (1). Reverse transcription-quantitative polymerase chain reaction (RT-qPCR) has been widely used to qualitatively and quantitatively determine SARS-CoV-2 gene targets (2). However, the construction of new fixed molecular diagnostic laboratories takes time. This delay affects the prevention and control of SARS-CoV-2. Thus, a mobile biosafety laboratory (MBS-Lab) is urgently needed to cope with emergent epidemics.

MBS-Lab was first used in northern Australia during a melioidosis outbreak in 1997 (3). At that time, only a simple sample collection kit and light microscope were inside the lab. With the development of MBS-Lab, the fully self-reliant vehicles are equipped with the latest molecular diagnostic and biocontainment equipment (4, 5). Mobility is the main characteristic of MBS-Labs as it enables quick on-site screening. Many research and development centers have created MBS-Labs of various biosafety levels, including P2, P3, and P4. Recently, many MBS-Labs have been used for epidemiological research, infectious disease control and other health emergencies. From March 2014 to March 2015, a unit of the European consortium used the European Mobile Laboratory to diagnose Ebola virus disease and malaria in Guéckédou, Guinea (6). Starting in October 2017, the Praesens Foundation developed an all-terrain MBS-Lab and tested it in Senegal for 6 months under various ecological conditions, demonstrating the capability for effective field diagnostics. The MBS-Lab and staff were deployed to manage a dengue outbreak in Louga city from 25 October to 23 November 2017 (7). Therefore, the MBS-Lab can be a novel solution assisting in rapid disease outbreak response and monitoring.

In this study we aimed to evaluate the detection capability and safety of MBS-Lab in dealing with SARS-CoV-2 pandemic response. A total of 236,717 samples from high-risk groups and the general population were tested. All staff strictly followed safety precautions to reduce exposure risks. In the end, no staff were infected with SARS-CoV-2 virus or other pathogens. Thus, the MBS-Lab may play a significant role in SARS-CoV-2 outbreak response through offering accurate and timely diagnostics.

## Materials and Methods

### Ethical Considerations

Ethical approval to conduct the study was provided by the First Hospital of Jilin University Ethics Committee (2021-296) and all participants had signed an informed consent form.

### Laboratory Biosecurity

The MBS-Lab included a biosafety cabinet with a high-efficiency particulate air filter. The classification of the biosafety cabinet including class II was listed in Table 1. The Class II type B biosafety cabinets have hard-ducted and vent outdoors, Class II type A2 biosafety cabinets vent outdoors, and Class II type A1 biosafety cabinets vent indoors. The Class II type A (A1 and A2) biosafety cabinets have 70% airflow recirculating, Class II type B1 biosafety cabinets have 30% airflow recirculating, while Class II type B2 biosafety cabinets have 0% airflow recirculating (8). In MBS-Lab, Class II type A2 biosafety cabinet was used. The above conditions provided a safe environment for handling pathogens. Moreover, there was an ultraviolet germicidal lamp inside the biosafety cabinet, an ultraviolet disinfection lamp on the ceiling and mobile sterilization car in every area and corresponding buffer room. There was an autoclave (>121°C and >205 kPa) in the corresponding buffer room of area II. The samples and personal protective equipment were autoclaved to avoid contamination of personnel and the environment. In addition, fumigation with hydrogen peroxide (H_2_O_2_) was regularly conducted within the truck and the workspace of the MBS-Lab. Moreover, all staff who worked in the MBS-Lab wore level 3 personal protective equipment (PPE), including work clothes, N95 protective masks, disposable hats, goggles, gloves, disposable shoe covers, face shields, medical protective clothing and disposable operation gowns. Other biosecurity measures include strengthening laboratory management, carrying out biosafety training, strengthening the protection awareness of staffs, developing specific standard operating procedures, establishing risk assessment and contingency procedures, monitoring fever or symptoms of staffs daily. Staffs were trained to have an appropriate sequence for safely donning and doffing PPE and practiced hand hygiene procedures. The disposal of laboratory waste was autoclaving and soaking with appropriate disinfectants. ultraviolet or air sterilizers were implemented in biosafety cabinets, every area and corresponding buffer rooms.

**Table 1:** Classification of the biosafety cabinet.

**Class**	**Type**	**Protection**	**Open mode**	**Inflow velocity**	**Airflow**	**Exhausted air**
I	N/A	Personnel environment	Open front	Negative pressure	0% recirculating	Exhausts through HEPA filter to room or outdoors
II	A1	Personnel environment sample	Open front	75 ft/min	70% recirculating	Vent indoors
	A2			100 ft/min	70% recirculating	Vent outdoors
	B1			100 ft/min	30% recirculating	Hard-ducted vent outdoors
	B2			100 ft/min	0% recirculating	Hard-ducted vent outdoors
III	N/A	Personnel environment sample	Enclosed	100 ft/min	0% recirculating	Hard-ducted vent outdoors

### Study Setting

This study was carried out in eight mobile biosafety laboratories in Dongchang District. Before the official start, all staff received appropriate training, including (1) the standard operation procedure for entering and leaving the laboratory, (2) biosafety practices and procedures, (3) preventive measures for occupational exposure, (4) risk evaluation and precautions, and (5) data analysis and material management. The maintenance technicians and drivers also received job-specific training. Each team had five staff members. Area I accommodated one staff member, area II accommodated two staff members and area III accommodated one staff member. The last member worked as a liaison and was responsible for communication between the laboratory and external staff.

### Study Population and Sampling

The study population included both high-risk groups and the general population. High-risk groups refer to exposure and proximity to the confirmed COVID-19 cases. This study included male and female of all ages who provided written informed consent to participate. However, for children under 18 years of age, parents or legal guardians signed the consent forms. After collecting on-site samples, we combined the samples from 10 individuals or one family in a single test tube for the general population. For high-risk groups, single samples rather than mixed samples were used.

### Mobile Biosafety Laboratory Workflow

#### Reagent Preparation Area

**Figure 1:**
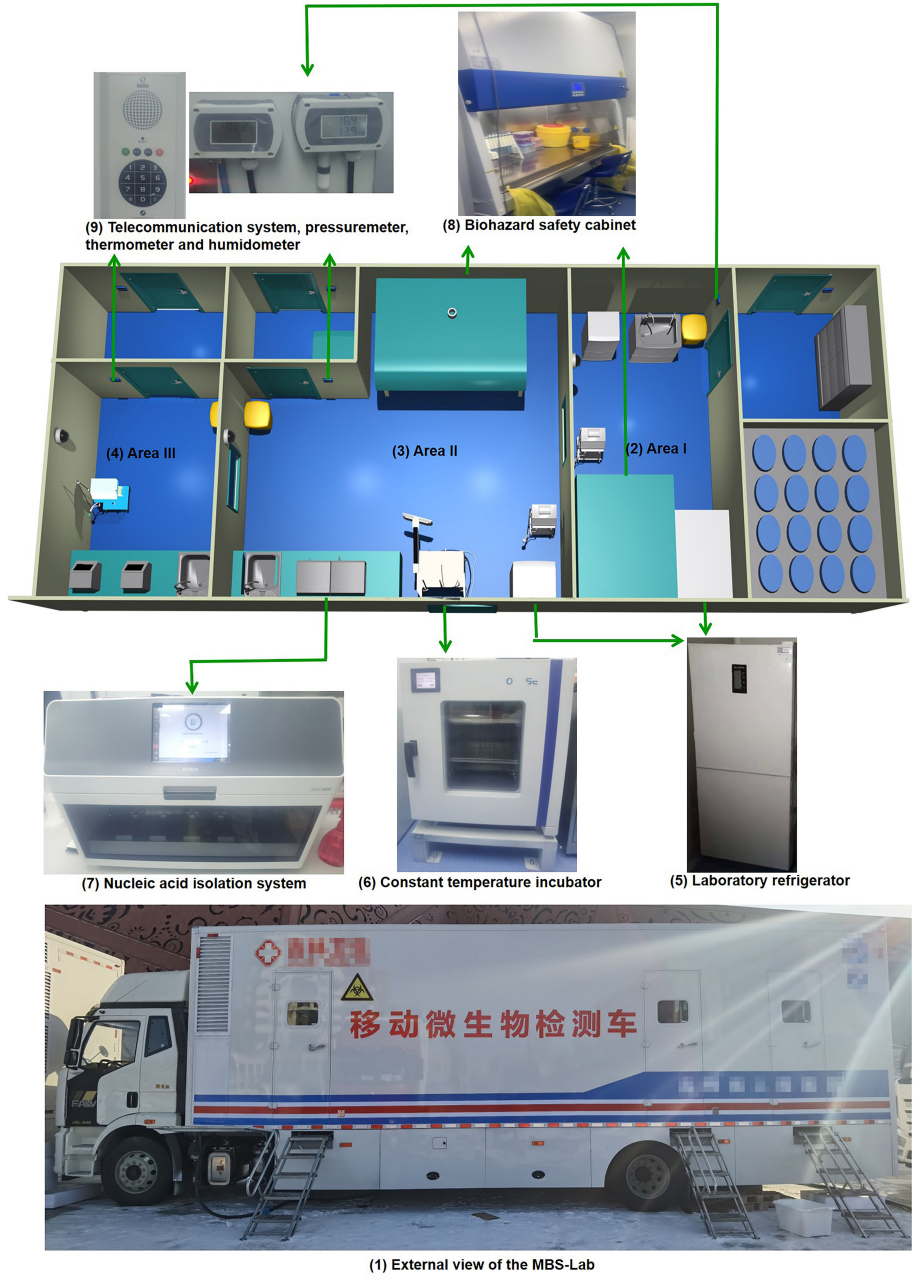
Layout of main devices in the mobile biosafety laboratory. (1) External view; (2) area I; (3) area II; (4) area III; (5) laboratory refrigerator; (6) constant-temperature incubator; (7) nucleic acid-isolation system; (8) biohazard safety equipment; (9) telecommunication system, pressure meter, thermometer and hygrometer.

The reagent preparation area is also called area I. It contained a biosafety cabinet, a pressure meter, a thermometer, a hygrometer, a telecommunication system and a biohazard waste container. A 4°C refrigerator was used for reagent storage. In addition, a −20°C freezer was used to store frozen reagents (Figure 1).

The Nucleic Acid Extraction Kit (Lot 510160, Zybio, Chongqing, China) was used to extract the SARS-CoV-2 RNA *in vitro* using a magnetic bead method. The Nucleic Acid Extraction Kit includes extraction reagent I, extraction reagent II, the elution buffer, proteinase K and the magnetic beads solution. If any crystals were present in extraction reagent I, the reagent was not used until they were fully dissolved. The aluminum wrapping film was removed from extraction reagent I, extraction reagent II and the elution buffer. The magnetic rod sleeves in the 96-well reagent kit were filled with magnetic beads. Proteinase K was mixed well to prepare it for use, and 15 μL was added to each well in the extraction reagent I plate. After thawing at room temperature, the components were mixed by oscillation and centrifuged at 8,000 rpm for several seconds before use. All reagents were unpacked, dissolved at room temperature and transferred to area II through the delivery window.

#### Specimen Preparation Area

The specimen preparation area is also called area II. It contained a biosafety cabinet, pressure meter, thermometer, hygrometer, telecommunication system, constant-temperature incubator, nucleic acid-isolation system and a biohazard waste container. Area II was equipped with a 4°C refrigerator for reagent storage and a −20°C freezer for frozen reagents (Figure 1).

In this area, the main operations were receiving samples, extracting nucleic acids and adding them to PCR tubes. The Detection Kit (Lot 2021007, Da'an, Guangzhou, China) was used to qualitatively detect the SARS-CoV-2 ORF1ab and N genes using a RT-qPCR assay. The Detection Kit includes the SARS-CoV-2 PCR reaction solution A, reaction solution B and positive and negative controls. After thawing at room temperature, the components were mixed by oscillation and centrifuged at 8,000 rpm for several seconds before use. PCR reaction solution A (17 μL) and PCR reaction solution B (3 μL) were added to each PCR tube. We used the nucleic acid-isolation system EXM6000 (Zybio Inc., Chongqing, China) to automatically isolate and purify the SARS-CoV-2 RNA.

#### Amplification Detection Area

The amplification detection area is also called area III. It included a pressure meter, thermometer, hygrometer, telecommunication system, amplification instrument, computer and biohazard waste container (Figure 1).

In this area, the main operations were nucleic acid amplification and analysis of the products. We used the amplification instrument Gentier 96E/96R (Xi'an Tianlong Science and Technology Development Co., Ltd., Xi'an, China) for amplification.

### Testing Report and Analysis

The positive control is pseudovirus containing 2019-nCoV target fragments and 2019-nCoV internal control gene fragments (RNase P gene). The negative control is 2019-nCoV internal control gene fragments (RNase P gene). Negative controls required no Cq (quantification cycle) values or obvious amplification curve for *N* gene and *ORF1ab* gene and a Cq ≤ 25 for internal control gene. Positive controls required Cq-values ≤ 22 for *N* gene and *ORF1ab* gene. The above requirements were applied at the same time for each experiment.

Results were considered negative when there was a Cq-value > 30 or no Cq-value for *N* gene, a Cq-value > 30 or no Cq-value for *ORF1ab* gene and a Cq-value ≤ 30 for internal control gene. Results were considered positive when there was a Cq-value ≤ 30 for *N* gene and a Cq-value ≤ 30 for *ORF1ab* gene with no amplification curve for internal control gene. A retest was required for a Cq-value ≤ 30 for *N* gene and a Cq-value > 30 or no Cq-value for *ORF1ab* gene with or without an amplification curve for internal control gene. A retest was also required for a Cq-value > 30 or no Cq-value for *N* gene and a Cq-value ≤ 30 for *ORF1ab* gene with or without an amplification curve for internal control gene. In addition, a retest was required for a Cq-value > 30 or no Cq-value for *N* gene and a Cq-value > 30 or no Cq-value for *ORF1ab* gene with or without an amplification curve for internal control gene. If the retest result of *N* gene or *ORF1ab* gene was positive (Cq-value ≤ 30) and internal control gene was positive (Cq-value ≤ 30), the specimen was considered positive for SARS-CoV-2. If the retest results of *N* gene and *ORF1ab* gene were both negative (Cq-value > 30 or no Cq-value) and internal control gene was positive (Cq-value ≤ 30), the specimen was considered negative for SARS-CoV-2. If the retest results of *N* gene, *ORF1ab* gene and internal control gene were all negative (Cq-value > 30 or no Cq-value), the sampling and testing processes were repeated.

### Staff Members and Worksite Layout

The team contained seven staff: one external contact, one infection control practitioner, one dedicated driver and four scientists, each engaged in specialized tasks (Table 2). One member worked as an external contact and was in charge of communications between staff in the laboratory and external staff. He or she coordinated between the local community and the laboratory, with roles including sample reception, data analysis and release of results.

**Table 2:** Overview of the mobile biosafety laboratory diagnostic team and tasks.

**Team role**	**Number of staff**	**Tasks**
External contact	1	Communication between laboratory and external staff, sample reception, data analysis and release of results
Scientists	1	Area I (reagent preparation, recordkeeping, checking reagents, and consumable supplies)
	2	Area II (sample reception, serial numbering of samples, sample dosing, RNA extraction, storage of positive and negative controls, recordkeeping)
	1	Area III (nucleic acid amplification and analysis)
Infection control practitioner	1	Supervision and guidance to ensure that safety standards were strictly followed, monitoring the health condition of each staff member and assessing the risk of infection
Driver	1	Stabilizing the vehicle, setting up the laboratory, sterilization of lab trash, solving minor technical issues related to the vehicle and equipment, ensuring water and electricity supply and security of the environment around the laboratory

The staff member in area I filled in records including the humidity and temperature of area I, the refrigerator temperature and the usage of the biosafety cabinet and checked reagents and the supply of consumables. The staff members in area II kept records of daily experimental processes.

After completing each round of testing, cleaning of the laboratory was performed. Each day, MBS-Lab, PPE and the equipment inside were sterilized for three times. Instrument surface, floor and table was wiped and disinfected with preparing fresh 2,000 mg/L chlorine for 30 min each time. Then, chlorine was subsequently removed as it is caustic and may damage equipment. Seventy percentage Alcohol was used to wipe down the metal surfaces (9). In addition, the ultraviolet lamps in the biosafety cabinet, area II and the corresponding buffer room were turned on for 1 h in order to disinfect the air. The infection control practitioner monitored, observed and guided the implementation of the core infection control system. In the end, a 6 h sufficient formaldehyde stifling had diffused to sterilize the lab thoroughly.

### Quality Control

The detection of SARS-CoV-2 nucleic acid requires extreme detection accuracy. Quality control is of great importance. Thus, it is necessary to establish a system of documentation to ensure SARS-CoV-2 nucleic acid testing results accurate. In China, we followed the principle of ISO 15,189 standard, including quality manuals, procedure files, operation instructions, and record forms. In addition, two kinds of external quality assessment (EQA) program were participated, China's Ministry of Health and Jilin province. Two positive controls and two negative controls were done every batch. Inter-laboratory comparison is with the nucleic acid testing base of the Central Hospital of Tonghua City and the First Hospital of Jilin University.

## Results

### Epidemiology

The infection began to spread from an asymptomatic carrier for products sale in Dongchang District, Tonghua City, Jilin Province, China. The asymptomatic carrier called together many old people. According to the Notification of Tonghua Municipal Health Committee, the number of confirmed cases in Tonghua City grew to 123, including 30 cases with light type, 83 cases with common type, 7 cases with severe type, 3 cases with critical type until 20 January 2021. On Jan 18 the small city is in lockdown. At that time, the first round was completed by the nucleic acid testing base of the Central Hospital of Tonghua City. Then, the number of daily new cases started to increase. As of March 3, 2021, 320 laboratory confirmed COVID-19 cases with only 1 death.

### Performance of the Mobile Biosafety Laboratory

The lab was powered by lithium-ion batteries. The batteries could be charged by a diesel generator and the local electrical grid, with automatic switching to ensure reliable and real-time power supply. In the event of a sudden power outage, a dedicated uninterruptible power supply was able to support experimental instruments, automatic control systems, illumination and ventilation for at least 45 min. The overall power supply system guaranteed the proper functioning of the laboratory. In addition, the MBS-Lab was equipped with an internal communications network and achieved safe communication channels between staff. There was also a video surveillance system. The status and data such as temperature, humidity, pressure, power system monitoring, local time, test reports and error logs were displayed on computer screens. The doors were interlocking. The setup allowed screening of suspected SARS-CoV-2 cases within 4 h after sample reception, while providing protection for humans, specimens and the environment.

The laboratory was equipped with an advanced ventilation system. The ventilation system used 100% fresh air purified by filters and the air renewal rate was up to 25 times per h. In addition, the pressure was reduced step-by-step, for instance, 15 Pa in area I, 10 Pa in the area I buffer room, −5 Pa in the area II buffer room, −10 Pa in area II, −10 Pa in the area III buffer room and −15 Pa in area III. This cascade of low pressure ensured that the air flow was unidirectional from the outside to the inside. The air cleanliness class was seven inside the lab.

### Biosafety Risk Management

**Figure 2:**
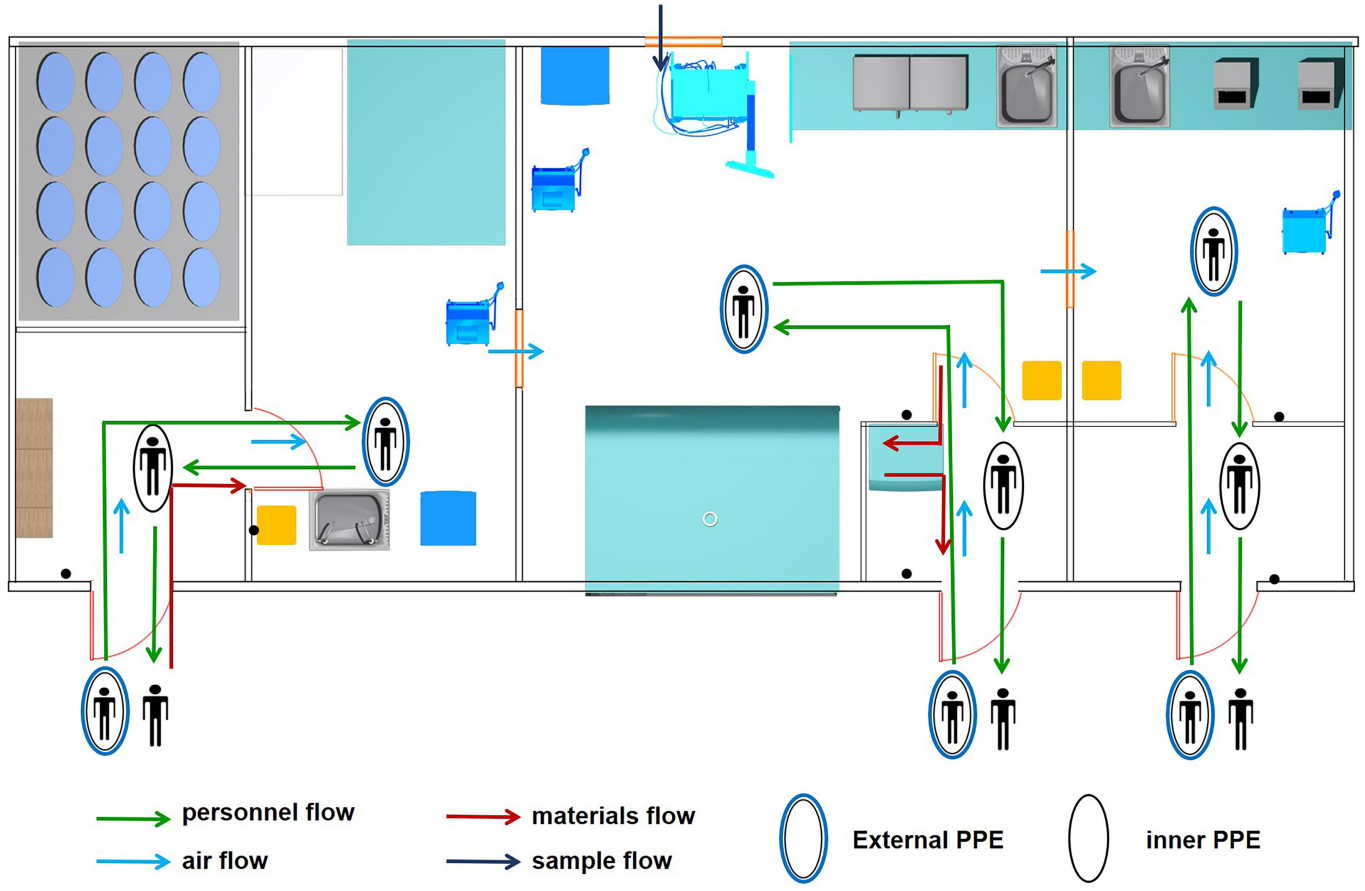
Four flows involved in biosafety risk. Personnel, air, sample and material flows were the main components of biosafety risk in the mobile biosafety laboratory.

The MBS-Lab was specifically designed to handle SARS-CoV-2. It was able to protect humans and the environment from exposure to the virus due to the safety equipment, facility design and laboratory practices. There were four flows (personnel, air, samples and materials) involved in the biosafety risk (Figure 2).

Under normal conditions, at most two staff worked in each room of the MBS-Lab to avoid affecting air flow. Before entering the MBS-Lab, all staff wore level 3 protection PPE. After finishing their tasks, staff took off their PPE layer by layer to reduce contamination. Furthermore, a seven-step hand-washing method was strictly followed. Staff then showered off-premises.

Samples were brought into area II through the external sample hatch with a pressure of −10 Pa. Other sterile materials were brought in through the personnel flow. Lab waste was transmitted to the area II buffer room and autoclaved using indicator tape.

### Laboratory Testing Results

There were in all four rounds related to SARS-CoV-2 nucleic acid screening. The first round was completed by the nucleic acid testing base of the Central Hospital of Tonghua City. Because of the excessive amount of work, the MBS-Lab and a trained diagnostic team were dispatched to assist with screening and diagnosis on 17 January 2021 and joined in starting at the second round. The SARS-CoV-2 nucleic acid screening results are presented in Table 3.

**Table 3:** Samples and test results from 28 September to 11 November 2014.

**Round**	**Date**	**Single samples** ** tested/positive(%0)**	**Mixed samples (10 in** ** 1 or one family in** ** one) tested/positive (%0)**	**Resamples** ** tested/positive (%)**	**Total samples** ** tested**	**Total persons** ** tested**
Second	20 January 2021 to 22 January 2021	1,923/2 (1.04%0)	3,647/3 (0.82%0)	30/1 (3.33%)	5,600	38,393
Third	25 January 2021 to 27 January 2021	18/0 (0)	47,473/10 (0.21%0)	30/4 (13.33%)	47,521	102,676
Fourth	29 January 2021 to 31 January 2021	0/0	40,831/0(0)	0/0	40,831	95,648

In the second round, 5,600 samples from the high-risk groups and the general population were tested from 20 to 22 January 2021. Two single samples and three mixed samples (10 in 1) were identified as positive. By resampling from indicated patients again, 3 mixed samples (10 in 1) yielded 30 single samples. Thirty single samples were tested and only one was identified as positive. In the third round, 47,521 samples were tested from 25 to 27 January 2021. All 18 single samples were identified as negative while 10 mixed samples (one family in one) were identified as positive. By resampling again, the 10 mixed samples (one family in one) yielded 30 single samples. These 30 single samples were tested, and four cases were identified as positive. In the fourth round, 40,831 samples were tested from 29 to 31 January 2021. All mixed samples (one family in one) were identified as negative. At the end of every round, the focus was turned to immediately isolating positive cases (Table 4). The positive cases were isolated and treated in hospital, avoiding visits family members, friends, colleagues and others.

**Table 4:** The Cq-value of positive samples.

**Positive samples**	**Cq-value**		
	**ORF1ab gene**	***N* gene**	**Internal control gene**
102422116721117	28.715	27.383	20.168
1018787901211F	27.473	25.246	20.074
10646744755809	26.184	28.418	20.231
104682244736074	27.452	23.510	20.027
10211801279300	28.745	27.301	20.340
12866101835273	24.523	26.401	20.052
10789430218546	26.770	28.553	20.131

## Discussion

In this study, a total of 93,952 samples were tested from 20 to 31 January 2021. The average sample turnaround for each round is 3 days. Three samples were positive in the second round, while four samples in the third round. There is no positive sample in the fourth round. The detectability and safety of MBS-Lab were evaluated in screening SARS-CoV-2 nucleic acids among high-risk groups and the general population. As an emergency facility for infectious disease control, the MBS-Lab satisfies the requirements of ports and other remote areas far from fixed laboratories and supplements the capabilities of fixed laboratories.

One limitation of our study is that we only tested one type of clinical sample. Testing of samples from multiple sites, including bronchoalveolar lavage fluid, nasal swabs, pharyngeal swabs, sputum, fiber bronchoscope brush biopsies, feces or blood, can improve the sensitivity of RT-qPCR for the diagnosis of SARS-CoV-2 infection (10). This can reduce false-negative test results. To screen more individuals, we mixed 10 individual oropharyngeal swabs into one tube as one sample to test the general population. For high-risk groups, individual samples were tested.

Another limitation of the lab is lack of a −80°C freezer inside the lab. For short-term storage (within 1 day), samples were stored in a 4°C freezer in area II. However, for long-term storage, samples should be stored in a −80°C freezer. The samples were packaged carefully, and after surfaces were disinfected with 0.25% chlorine-containing disinfectant they were transported to the Central Hospital of Tonghua City nearby.

Screening test results are important for the management of both infected asymptomatic and symptomatic individuals. The isolation of positive cases can prevent human-to-human transmission of SARS-CoV-2. Two negative results for oropharyngeal swabs can be regarded as an indicator that isolation is not necessary. Thus, the sensitivity and specificity of the test results are crucial. Currently, RT-qPCR is the gold standard method for the diagnosis of SARS-CoV-2 (11). The primary analytical methods focus on quantitative responses and cycle number determination. The Cq-value is the point when the fluorescence intensity grows above the background level and crosses a predetermined threshold value. Our diagnostic algorithm has a suggested Cq-value of 30. However, false-positive or false-negative results might exist, where the true number of infected individuals is smaller or larger than the number of positive tests, respectively. False-positive results are mainly from contamination with other pathogens and exogenous or endogenous interfering substances. At that time, time was limited and the task is heavy. It was uncertainty when control materials from the College of American Pathologists (CAP) could get through China Customs. So, two kinds of external quality assessment (EQA) program were participated, China's Ministry of Health and Jilin province. In EQA of Jilin province, there were 5 controls (2021101, 2021102, 2021103, 2021104, 2021105) to detect *ORF1ab* and *N* genes. The results we examined were consistent with the expected results, 2021101, 2021103, 2021105 were positive and 2021102, 2021104 were negative. In EQA of China's Ministry of Health, there were 5 controls (202111, 202112, 202113, 202114, 202115). The results we examined were consistent with the expected results, 202111, 20212, 202113, 202114 were positive, and 202115 were negative. Thus, reagent preparation and sample dosing were conducted in the biosafety cabinet. In addition, areas I, II and III and the biosafety cabinet were exposed to ultraviolet radiation periodically to eliminate nucleic acid contamination. At this point, SARS-CoV-2 has spread extensively for nearly 1 year, and the virus is prone to mutations. Scientists have identified 198 filtered recurrent mutations in the SARS-CoV-2 genome. Moreover, three sites in the *ORF1ab* gene were characterized as having a particularly large number of recurrent mutations (>15 events) (12). Therefore, genetic mutation of SARS-CoV-2, especially in the *ORF1ab* or *N* genes, may lead to false-negative results. In order to investigate the mutation of *N* genes, 31,421 SARS-CoV-2 genome samples were collected on July 23, 2020. Through computing the mutation rate and mutation h-index, the authors have found that *N gene* is one of the most non-conservative genes in the SARS-CoV-2 genome. This study assume that *N gene* is particularly prone to mutations (13). In addition, RT-PCR is apt to show false-negative results during the incubation period and recovery phase. A retrospective study described 1,014 infected patients and found an estimated 41% false-negative rate with RT-PCR diagnostic tests (14).

There are four biosafety levels defined by the CDC (15). CDC has permitted to handle suspected or confirmed COVID-19 patient clinical specimens in BSL-2 facilities with enhanced work practices (16, 17). Thus, in specimen preparation area (area II), we used BSL-2 facilities with enhanced work practices, while in the reagent preparation area (area I) and amplification detection area (area III), we used BSL-2 facilities. Because all samples have been inactivated (18).

We collected oropharyngeal swabs rather than nasopharyngeal swabs to screen for SARS-CoV-2 in this study. The oropharyngeal swabs are less likely than the nasopharyngeal swabs to yield positive results (19). Luo et al. demonstrated that the sensitivity of oropharyngeal swabs was only 71% with one test, yet the sensitivity of oropharyngeal swabs reached 92.19% with a second round (20). Thus, to increase the sensitivity of oropharyngeal swabs for the rRT-PCR test, we conducted three rounds of screening within 10 days.

We mixed 10 oropharyngeal swabs in one tube for testing. In the second round of screening, three mixed samples were identified as positive; 10 mixed samples were identified as positive in the third round of screening. However, after resampling again, only one and four cases were identified as positive in the second and third rounds, respectively. Theoretically, if one mixed sample (10 in 1) is identified as positive, after resampling again, 10 single samples are tested, of which at least one should be positive. In other words, three mixed samples were identified as positive in the second round of screening; at least three of the 30 resampled single samples would be expected to be positive. Nevertheless, only one such sample was identified as positive, and the exact reasons for this are not known. Although relevant research is not available, we can speculate on possible causes. Due to the stage of infection, the viral load after resampling may be so low that the virus cannot be detected. Thus, further study is needed on the causes.

MBS-Labs have been successfully used to control Ebola virus disease and malaria in Guéckédou, Guinea (6), Ebola virus disease near Freetown, Sierra Leone, South Africa (4), a dengue outbreak in Louga city from 25 October to 23 November 2017 (7), whereas challenges still remain. The first challenge of MBS-Lab is lack high-level biosafety laboratories. No more than 70 BSL-4 laboratories have been established all over the world (21). Thus, the capacity of handle highly pathogenic microbes is limit. Secondly, due to the workplace is always in remote locations, there is no enough significant infrastructure to use, such as the refrigeration equipment, analysis equipment, sophisticated operational equipment. Thirdly, it is short of well-trained and experienced specialists. Although professional teams have been established in some countries, a relatively complete, independent management and professional group is in short supply in SARS-CoV-2 outbreak. In addition, the affairs of logistical requirements are another challenge, including accommodations, transportation into staffs, medication, food and clean water, access to electricity and security personnels (22).

As an emergency facility for infectious disease control, the MBS-Lab is characterized by high mobility and rapid on-site detection. It can detect the nucleic acids of SARS-CoV-2 and other pathogenic microorganisms. The MBS-Lab satisfies the requirements of ports and other remote far away from fixed laboratories for on-site inspection. Thus, the MBS-Lab acts as a key supplement to fixed laboratories. It is believed that the development prospects of the MBS-Lab will continue to improve, playing a greater role in disease control and prevention.

## Data Availability Statement

The original contributions presented in the study are included in the article/supplementary material, further inquiries can be directed to the corresponding authors.

## Ethics Statement

The studies involving human participants were reviewed and approved by Ethical approval to conduct the study was provided by the First Hospital of Jilin University Ethics Committee (2021). Written informed consent to participate in this study was provided by the participants' legal guardian/next of kin.

## Author Contributions

ZG, JH, and JX drafted the main manuscript, performed the data analysis, were responsible for guiding and supporting the experiments, and manuscript revisions. ZG, JH, JX, LL, and YS planned and performed experiments. ZG, JH, LL, and JX were responsible for experimental design. All authors contributed to the article and approved the submitted version.

## Conflict of Interest

The authors declare that the research was conducted in the absence of any commercial or financial relationships that could be construed as a potential conflict of interest.

## Publisher's Note

All claims expressed in this article are solely those of the authors and do not necessarily represent those of their affiliated organizations, or those of the publisher, the editors and the reviewers. Any product that may be evaluated in this article, or claim that may be made by its manufacturer, is not guaranteed or endorsed by the publisher.
